# F-doped TiO_2_ microporous coating on titanium with enhanced antibacterial and osteogenic activities

**DOI:** 10.1038/s41598-018-35875-6

**Published:** 2018-12-14

**Authors:** Jianhong Zhou, Bo Li, Yong Han

**Affiliations:** 1 Institute of Physics & Optoelectronics Technology, Baoji University of Arts and Sciences, Baoji, 721016 China; 2 State Key Laboratory for Mechanical Behavior of Materials, Xi’an Jiaotong University, Xi’an, 710049 China

## Abstract

To enhance bacterial resistance and osteogenesis of titanium (Ti) -based implants, TiO_2_/calcium-phosphate coatings (TiCP) doped with various amounts of fluorine (F) (designated as TiCP-F1, TiCP-F6, and TiCP-F9) were prepared on Ti by micro-arc oxidation. The F doped TiCP coatings possess a microporous structure (pore size of 3–4 μm in average diameter) which is evenly covered by nano-grains of 30–60 nm in size. Successful F incorporation into TiCP was determined by X-ray photoelectron spectroscopy, and it shows weak influence on the microstructure, phase compositions, surface roughness and wettability of TiCP. All the coatings bonded firmly to the Ti substrates and showed enduring high adhesion strength in biological circumstances. The bacterial resistance and osteogenesis of the coatings were evaluated by implanting testing materials *in vitro* and in an infected rabbit model caused by bacteria. Both the *in vitro* and *in vivo* results indicated that TiCP and TiCP-F1 were of much higher osteogenic activity compared with Ti but lacking of bacterial resistance, whereas TiCP with high F addition (TiCP-F6 and TiCP-F9) exhibited both dramatically improved bacterial resistance and osteogenesis. In summary, TiCP-F6 possessed the best antibacterial and osteogenic activities, especially exhibited excellent osseointegration efficacy in the infected rabbit model.

## Introduction

Titanium (Ti) is widely applied in producing bone implants because of its high corrosion resistance, excellent biocompatibility, and good mechanical properties^1,2^. However, Ti is bioinert without antibacterial activity, the implant-related infection caused by bacteria^3,4^ and poor osseointegration of Ti^5^ will result in implantation failure. Hence, advanced Ti-based implants with dual functions of antibacterial ability and osteogenesis are stringently needed in medical treatment.

Loading and delivering of inorganic biological active elements shall be an effective way to enhance the antibacterial and osteogenic activities for Ti-based implant. The inorganic elements are quite stable to facilitate the incorporation process and usually functioned in very low doses. Thus, long-term antibacterial and osteogenic effects can be realized by regulating the loading contents and the release rate^6^ from Ti-based implant with limited reservoir. Regarding the inorganic bioactive element, fluorine (F) possesses not only excellent cytocompatibility but also good antibacterial ability^7,8^. Furthermore, it is worth noting that F is an essential trace element in human bone and plays an important role in regulating osteogenesis^8,9^. Our previous works have shown that F-doped TiO_2_ coating on Ti surface induced better antibacterial and osteogenic activities compared to the one without F^10^. However, some works have indicated that overdose of F ions inhibited the proliferation and osteogenic differentiation of osteogenesis-related cells^11,12^. Hence, optimizing F incorporation dose in the coating is essential.

Regarding the method for the inorganic element incorporation into the Ti-based implant surface, micro-arc oxidation (MAO) shall be a more feasible choice. MAO can form a rough, firmly adhering TiO_2_ coating on the Ti surface, which has been widely investigated to show enhanced bioactivity^13–15^. Meanwhile, MAO also provides an effective means to incorporate the inorganic elements such as calcium (Ca), phosphorus (P), strontium (Sr), and F into the TiO_2_ coating^10,15^. In the present study, TiO_2_/calcium-phosphate (TiCP) coatings doped with different amounts of F, namely TiCP-F1, TiCP-F6, and TiCP-F9, where the Arabic numbers represent the average content of F in the coatings, were developed on Ti by the MAO. Rabbit bone marrow stem cells (MSCs), *Escherichia coli* (*E. coli*), and *Staphylococcus aureus* (*S. aureus*) were employed to study the cytocompatibility, osteogenesis and antibacterial ability of the coatings, respectively. Moreover, the *in vivo* antibacterial and osteogenic activities of the coatings were studied in a bacterial-infected rabbit model. The present work will give rise to an advanced Ti-based implant with improved clinical performance.

## Results

### Characterization of the coatings

**Figure 1:**
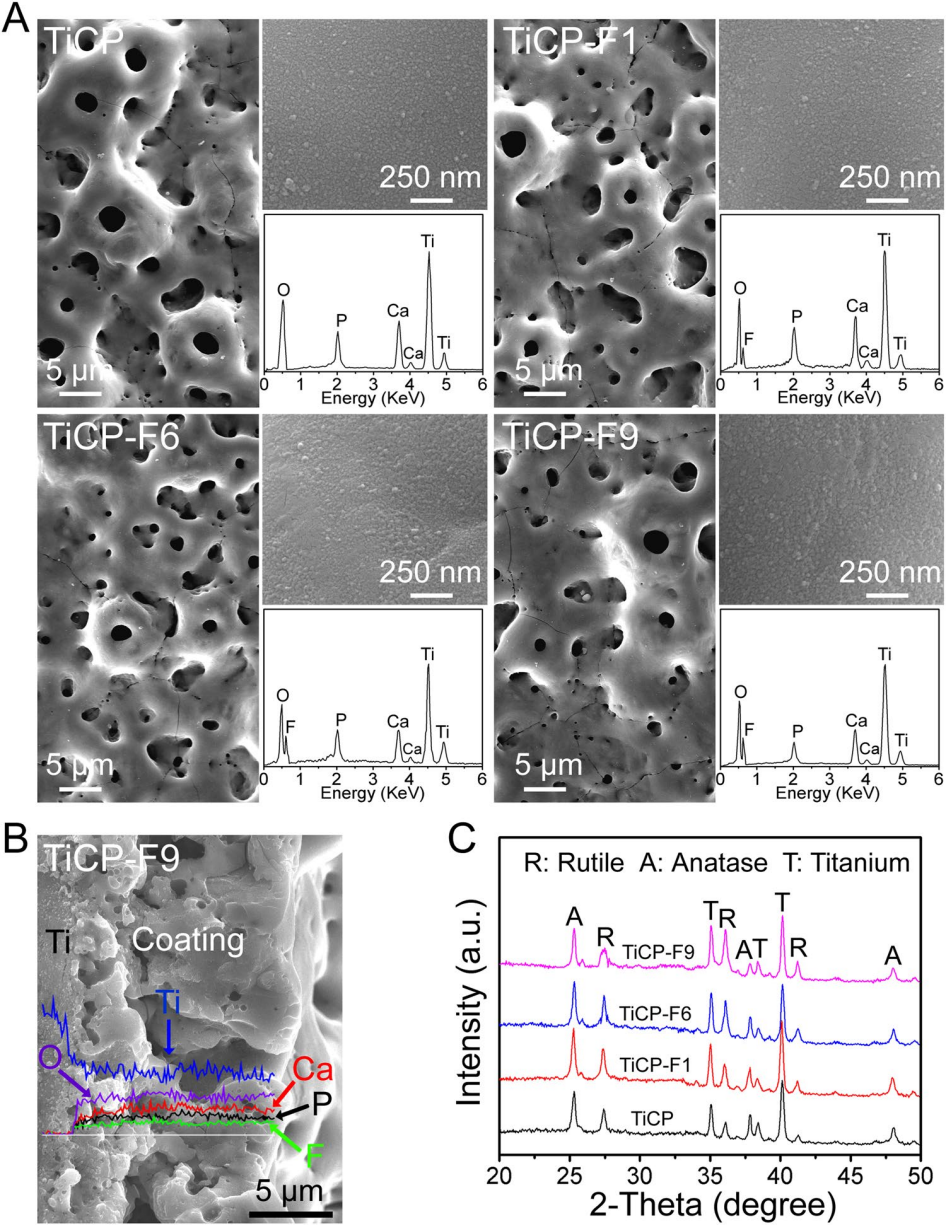
(**A**) SEM images of TiCP, TiCP-F1, TiCP-F6, and TiCP-F9 with EDX pattern and higher magnification image inserted, (**B**) Cross-sectional morphology and elemental profiles of TiCP-F9, (**C**) XRD patterns of the coatings.

Figure 1A shows that TiCP, TiCP-F1, TiCP-F6, and TiCP-F9 have similar typical microporous MAO structure, with micropores of an average diameter of 3–4 μm distributing homogeneously, and uniformly covered with the nano-grains of ~30–60 nm in size (top insets in Fig. 1A). The EDX results (bottom insets in Fig. 1A) show that only Ti, O, Ca, and P are detected in TiCP, while additional F can be further detected in TiCP-F1, TiCP-F6 and TiCP-F9. The surface elemental compositions detected by XPS (Table S1) indicate that the F contents in the coatings can be modulated by the NaF concentration in the MAO electrolytes, which shows a positive correlation. The elemental distribution on the cross-section of TiCP-F9 (Fig. 1B) also shows that the coating contains F except Ti, O, Ca and P, which further confirms the successful incorporation of F in the coating. There is no discontinuity at the interface of the coating/Ti substrate (Fig. 1B), exhibiting a firm binding of the coating to the Ti substrate. The XRD patterns (Fig. 1C) show that all the coatings consist of predominant anatase and rutile TiO_2_, and no feature peaks of F-containing compounds are detected in any coatings.

**Figure 2:**
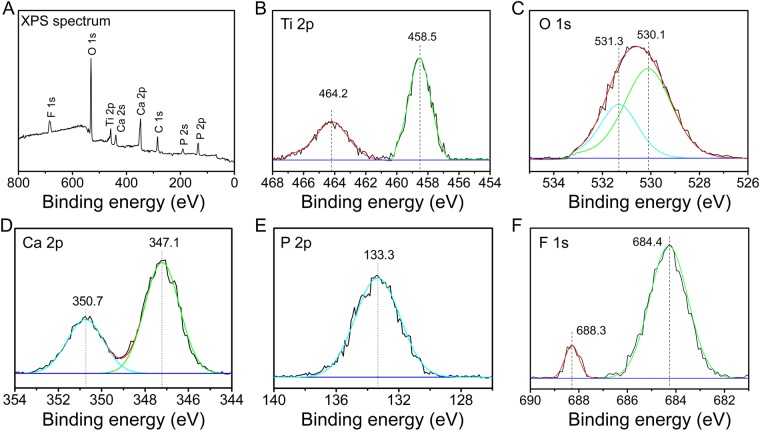
XPS spectrum (**A**) and high-resolution spectra of Ti2p (**B**), O1s (**C**), Ca2p (**D**), P2p (**E**), and F1s (**F**) detected from the surface of TiCP-F9.

The XPS full spectrum obtained from TiCP-F9, as a representative of the F incorporated coatings, is shown in Fig. 2A. Besides the feature peaks of Ti, O, Ca, and P, the feature peaks of F are also detected, again confirming the successful F incorporation in TiCP-F9. The high-resolution spectra of the coating are shown in Fig. 2B–F. The Ti2p spectrum corresponds with typical binding energies for TiO_2_^16^. The O1s spectrum is deconvoluted into two Gaussian component peaks. The peak located at 530.1 eV is assigned to O1s in TiO_2_^17^, and the other peak at 531.3 eV corresponds with O1s in P=O- groups (Ca_3_(PO_4_)_2_ or CaHPO_4_^18^. The Ca2p peaks are located at 347.1 eV and 350.7 eV, and the P2p peak is located at 133.3 eV, which indicate that the Ca2p and P2p exist in the form of calcium phosphate phases (such as α-tricalcium phosphate, amorphous calcium phosphate) in the detected surface layer^19^. The F1s peaks are located at 684.4 eV and 688.3 eV, indicating that a part of the incorporated F exists in the form of Ti-F and the other part goes into the lattice of TiO_2_^20,21^.

The roughness and wettability of TiCP, TiCP-F1, TiCP-F6, and TiCP-F9 were measured and listed in Table S2. The results show that these coatings have similar submicroscale roughness, evaluated by the average roughness (Ra), root-mean-square roughness (RMS), and selection of 10-point height of irregularity roughness (Rz). Meanwhile, the water contact angles on the coatings are also very similar. Totally, the F incorporation does not significantly change the roughness and wettability of the coating surface.

### Ion release and adhesion strength of the coatings to the substrates

**Figure 3:**
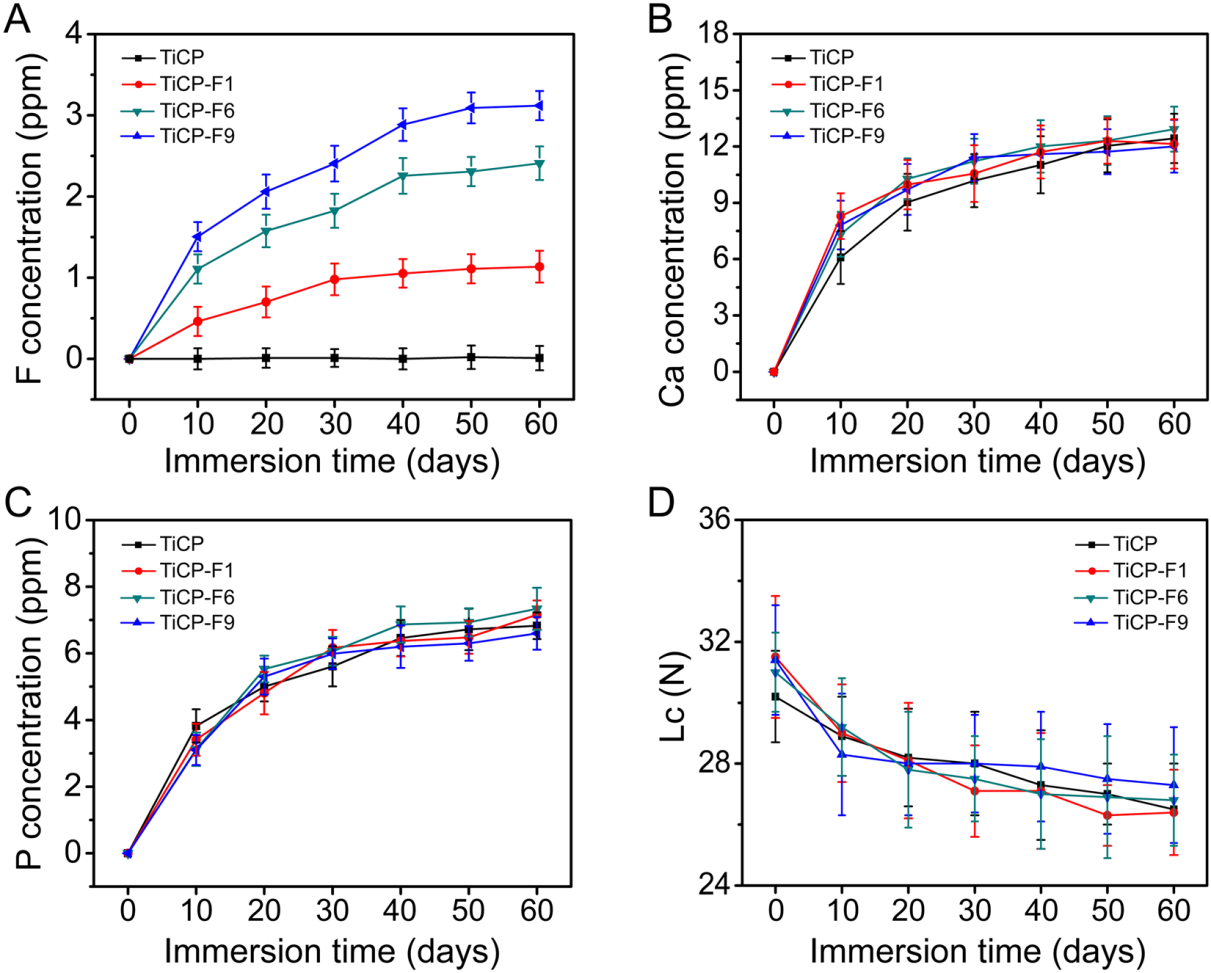
Cumulative release profiles of (**A**) F, (**B**) Ca, and (**C**) P from the coatings into physiological saline solutions, and (**D**) adhesion strength of the coatings before and after immersion in physiological saline solutions of different durations.

The release of ions as well as the adhesion strength of the coatings to the substrates after immersion in physiological saline solutions (PS solutions, e.g., 0.9 wt% NaCl aqueous solutions) of different durations are shown in Fig. 3. TiCP can only release the Ca and P ions, while TiCP-F1, TiCP-F6, and TiCP-F9 can release the additional F ion. The release of the F, P, and Ca ions increases commensurately with the soaking duration, suggesting a constant release mode. For the F ion release, there is a controlled release profile. The released F dose is positively correlated with the incorporated F amount in the coatings, following the order of TiCP-F9>TiCP-F6>TiCP-F1 (Fig. 3A). In addition, the F incorporation and release does not obviously influence the release profiles of Ca and P ions (Fig. 3B,C).

The firm bonding between the coating and the substrate is essential to realizing the long-term function of the implants, without which the debris delaminated from the coatings would lead to aseptic loosening-derived implants failure. Figure 3D shows the adhesion strength between the coatings and Ti substrates after immersion in PS solutions for different durations. The Lc values of all the coatings without immersion are about 29.5 N in average, suggesting a strong and firm bonding between the coatings and the Ti substrates, which is independent of the F incorporation. After immersion in PS solutions for as long as 4 weeks, the adhesion strength of the coatings retain well with slight decrease, indicating long-term stability adhesion strength between the coatings and Ti substrates during usage in biological environment.

### *In vitro* antibacterial activity

**Figure 4:**
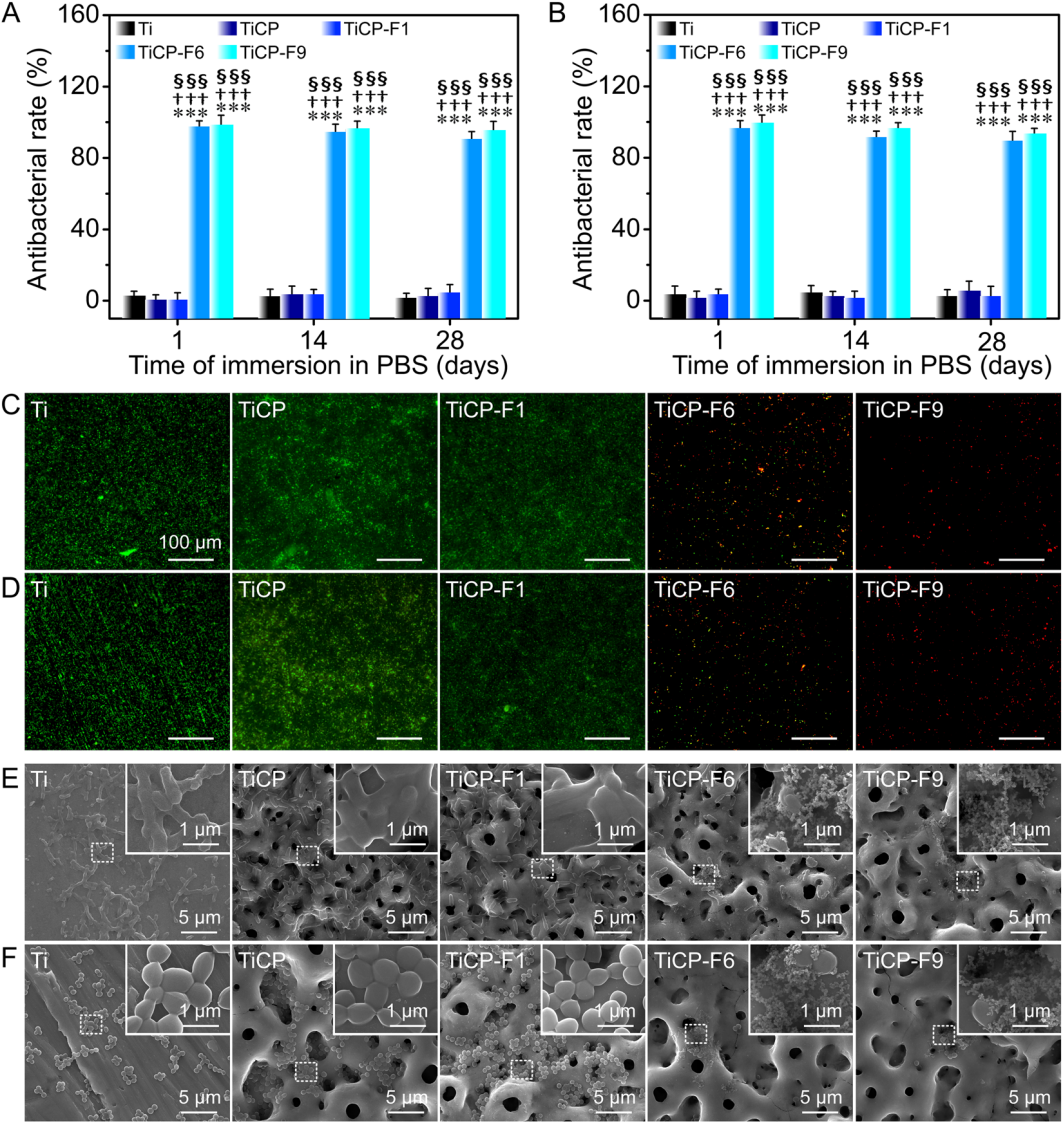
Antibacterial rates of the coatings as well as Ti after immersion in PBS for 1, 14 and 28 days against (**A**) *E. coli* and (**B**) *S. aureus*; Fluorescence images of adhered (**C**) *E. coli* and (**D**) *S. aureus* on the coatings as well as Ti after immersed PBS for 28 days, the dead bacteria appear red while the live ones are green. SEM images of (**E**) *E. coli* and (**F**) *S. aureus* incubated for 12 h on Ti and the coatings after immersed PBS for 28 days, with the corresponding higher magnification images of the circled regions inserted. Data are presented as the means ± SD, n = 4. ***p < 0.001 compared to Ti; ^†††^*p* < 0.001 compared to TiCP; ^§§§^*p* < 0.001 compared to TiCP-F1.

Figure 4A,B show the antibacterial activity of the coatings as well as Ti after immersion in PBS for 28 days against *E. coli* and *S. aureus*, respectively. The result indicated that Ti, TiCP and TiCP-F1 did not show any antibacterial efficacy against the bacteria at any time, suggesting that the Ca, P and low dosage of F ions released from the coatings have no ability to kill the bacteria. Noticeably, the antibacterial rates of TiCP-F6 and TiCP-F9 against the bacterial species were as high as about 97%, and which was no discernible decrease up to day 14. Even at day 28, TiCP-F6 and TiCP-F9 still maintained high antibacterial rates of at least 94%, suggesting long-term and effective antibacterial activity against *E. coli* and *S. aureus*.

Figure 4C,D show the long-term antibacterial activity of the coatings and Ti after immersion in PBS for 28 days by fluorescent staining assays. It is observed that there are a large number of viable (green) bacteria nearly unaccompanied by dead (red) ones on Ti, TiCP, and TiCP-F1, while rare viable bacteria can be seen on TiCP-F6 and TiCP-F9, indicating that have the function of effectively killing adhesive bacteria and preventing their colonization.

The number, morphology, and membrane integrity of bacteria on the coatings as well as Ti after immersion in PBS for 28 days were investigated by FE-SEM examination (Fig. 4E,F). A large number of bacteria can be seen on Ti, TiCP and TiCP-F1, while which is rare on TiCP-F6 and TiCP-F9. On Ti, TiCP and TiCP-F1, the *E. coli* are mostly in a rod morphology with abundant binary fission. However, on TiCP-F6 and TiCP-F9, very little intact bacteria are found while much completely lysed ones can be seen (Fig. 4E). On Ti, TiCP and TiCP-F1, the *S. aureus* revealed intact morphology and smooth surface (Fig. 4F), while on TiCP-F6 and TiCP-F9 nearly no intact bacterias but some bacteria debris can be found (Fig. 4F).

### Protein adsorption, cytotoxicity, cell adhesion, proliferation and cell morphology

**Figure 5:**
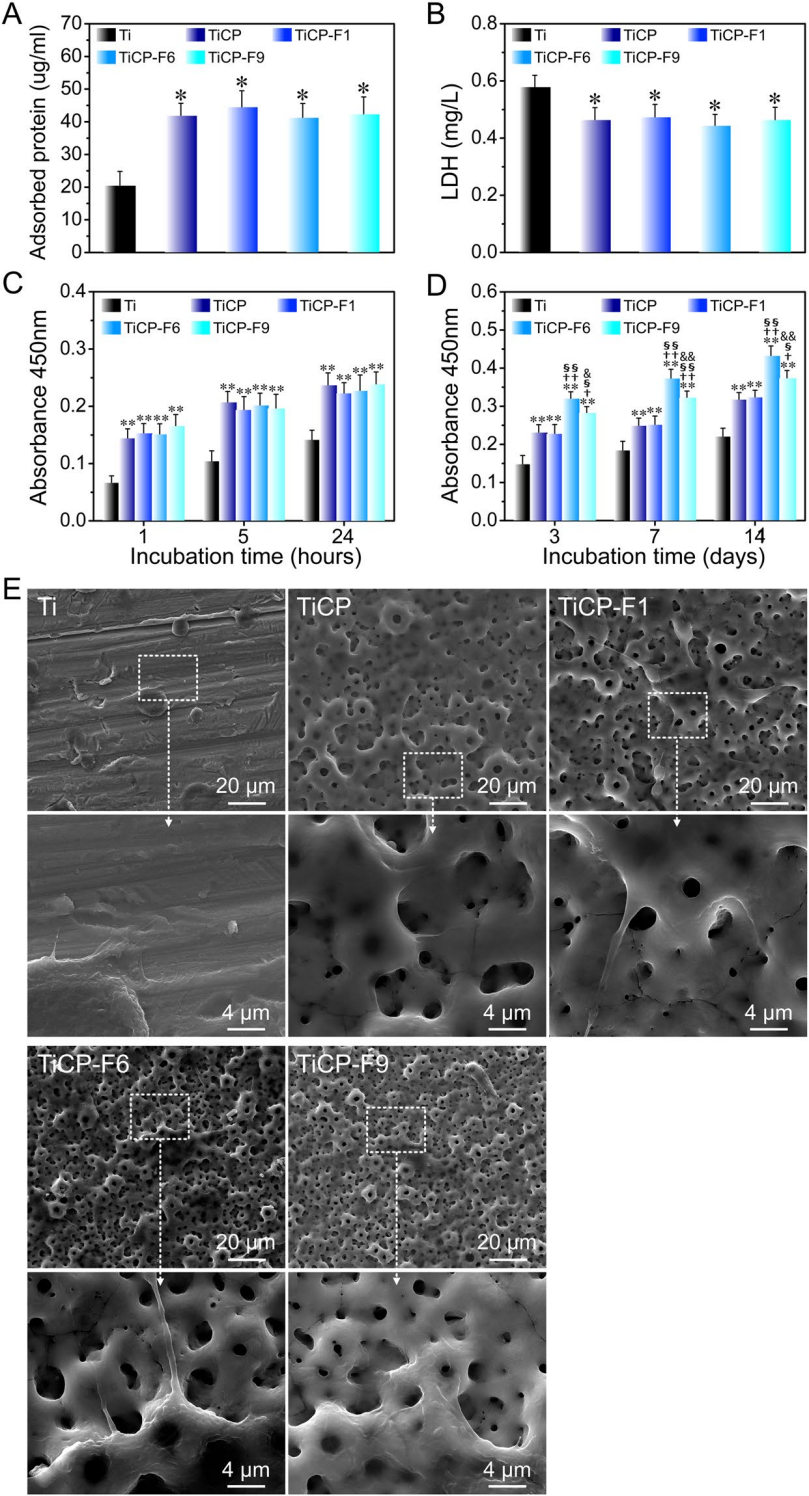
(**A**) Protein adsorption onto the coatings as well as Ti after 24 h of immersion in a-MEM containing 10% FBS, (**B**) LDH amount released by MSCs after 3 days of incubation, (**C**) MSCs adhesion measured by CCK-8 after 1, 5, and 24 h of culture, (**D**) MSCs proliferation measured by CCK-8 after 3, 7, and 14 days of culture, (**E**) SEM images of MSCs after culturing for 3 days on the coatings as well as Ti. Data are presented as mean ± SD, n = 5. *p < 0.05 and **p < 0.01 compared to Ti; ^†^*p* < 0.05 and ^††^*p* < 0.01 compared to TiCP; ^§^*p* < 0.05 and ^§§^*p* < 0.01 compared to TiCP-F1; ^&^*p* < 0.05 and ^&&^*p* < 0.01 compared to TiCP-F6.

The protein adsorption onto a material surface is considered to mediate the biological responses of the cells/tissues^22^. Hence, the amount of total protein adsorbed onto the coatings as well as Ti after 24 h of immersion in a-MEM containing 10% fetal bovine serum (FBS; Life Technologies, USA) was measured (Fig. 5A). In comparison with the Ti, TiCP, TiCP-F1, TiCP-F6, and TiCP-F9 gave rise to more adsorbed proteins, which may most probably be attributed to their rougher surfaces with larger surface areas for protein anchoring. While all the coatings did not induce apparent difference in the adsorbed protein amount, demonstrating that the incorporated inorganic elements have no obvious effect in this aspect.

The lactate dehydrogenase (LDH) released by cells cultured on the coatings was evaluated as an indication of cytotoxicity. As shown in Fig. 5B, TiCP, TiCP-F1, TiCP-F6, and TiCP-F9 exhibited no cytotoxicity compared with the Ti control. Hence, the F amounts released from the coatings are considered to be safe. Interestingly, the LDH release induced by the coatings was slightly smaller than that induced by the Ti control, indicating even enhanced cytocompatibility of the coatings, which is further supported by the subsequent *in vitro* cell adhesion and proliferation data.

Initial cell adhesion plays an important role of ensuring cell proliferation and differentiation on biomaterials^23^. The adhesion and proliferation of MSCs culturing on the coatings and Ti substrates were evaluated using cell counting kit-8 (CCK-8) assay. As shown in Fig. 5C, all the coatings led to more initial adherent cells compared with the Ti, due to their larger surface area and more protein adsorption. Similar initial adherent cell number can be seen among themselves, which demonstrats that the amount of the incorporated F in present study has no obvious effect on cell adherent. Generally, MSCs proliferate on all the coatings as well as Ti with time, while obvious difference in cell proliferation can be observed among them. MSCs proliferation after 3, 7, and 14 days of culture on the coatings and Ti show the general trend of TiCP-F6>TiCP-F9>TiCP-F1≈TiCP>Ti (Fig. 5D).

The cell shape on biomaterials is closely related to the cell functions^24^. The adhesion and spreading of MSCs after 3 days of culture on the coatings as well as Ti were observed by FE-SEM (Fig. 5E). The MSCs on Ti expand poorly with a spindle morphology, which is indicative of undifferentiated inactive cells. However, TiCP, TiCP-F1, TCP-F6, and TiCP-F9 can obviously improve the MSCs attachment and rendered them spread out extensively, covering or anchoring to the micropores on the surface (Fig. 5E). It can be inferred that the incorporation of F does not obviously influence the initial adhesion and spreading of MSCs compared with TiCP.

### *In vitro* osteogenic activity

**Figure 6:**
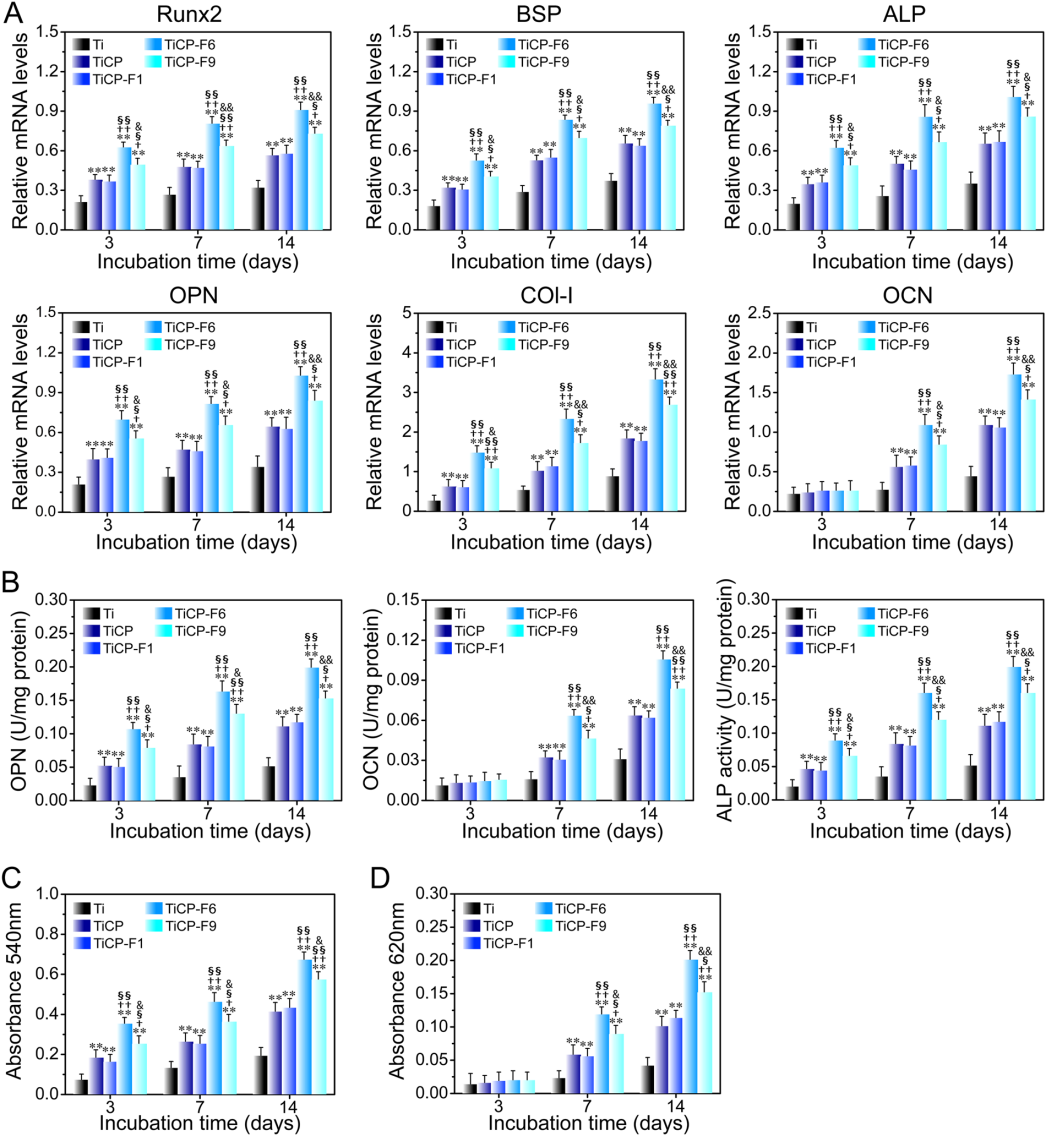
(**A**) Osteogenesis related gene expression of Runx2, BSP, ALP, OPN, Col-I, and OCN, (**B**) intracellular protein expression of OPN and OCN as well as ALP activity, and quantitative results of collagen secretion (**C**) and ECM mineralization (**D**) of MSCs incubated on the coatings as well as Ti for 3, 7 and 14 days. Data are presented as the means ± SD, n = 4. **p < 0.01 and ***p < 0.001 compared to Ti; ^†^*p* < 0.05 and ^††^*p* < 0.01 compared to TiCP; ^§^*p* < 0.05 and ^§§^*p* < 0.01 compared to TiCP-F1; ^&^*p* < 0.05 and ^&&^*p* < 0.01 compared to TiCP-F6.

The osteogenic differentiation of MSCs after cultured on the coatings as well as Ti was studied. The expressions of the osteogenesis-related genes including Runx2, BSP, ALP, OPN, OCN and type 1 collagen (Col-I) (Fig. 6A), the intracellular ALP activity, protein contents of OCN and Col-I (Fig. 6B), collagen secretion (Fig. 6C) and extracelluar matrix (ECM) mineralization (Fig. 6D) in MSCs after culturing on the coatings as well as Ti for 3, 7, and 14 days were measured. Totally, the gene and protein expressions of all the osteogenesis-related markers, collagen secretion and ECM mineralization all followed the rank of TiCP-F6 > TiCP-F9 > TiCP-F1≈TiCP > Ti. The results indicated that all the coatings could stimulate MSCs osteogenic-differentiation, which was obviously rely on the incorporated amount of F, however, it was not the more the better for the amount of incorporated F. Collectively, TiCP-F6 showed the best effect, but TiCP-F9 weakened this efficiency, possibly due to overdose F amount-derived side effect.

### *In vivo* antibacterial and osseointegration abilities

**Figure 7:**
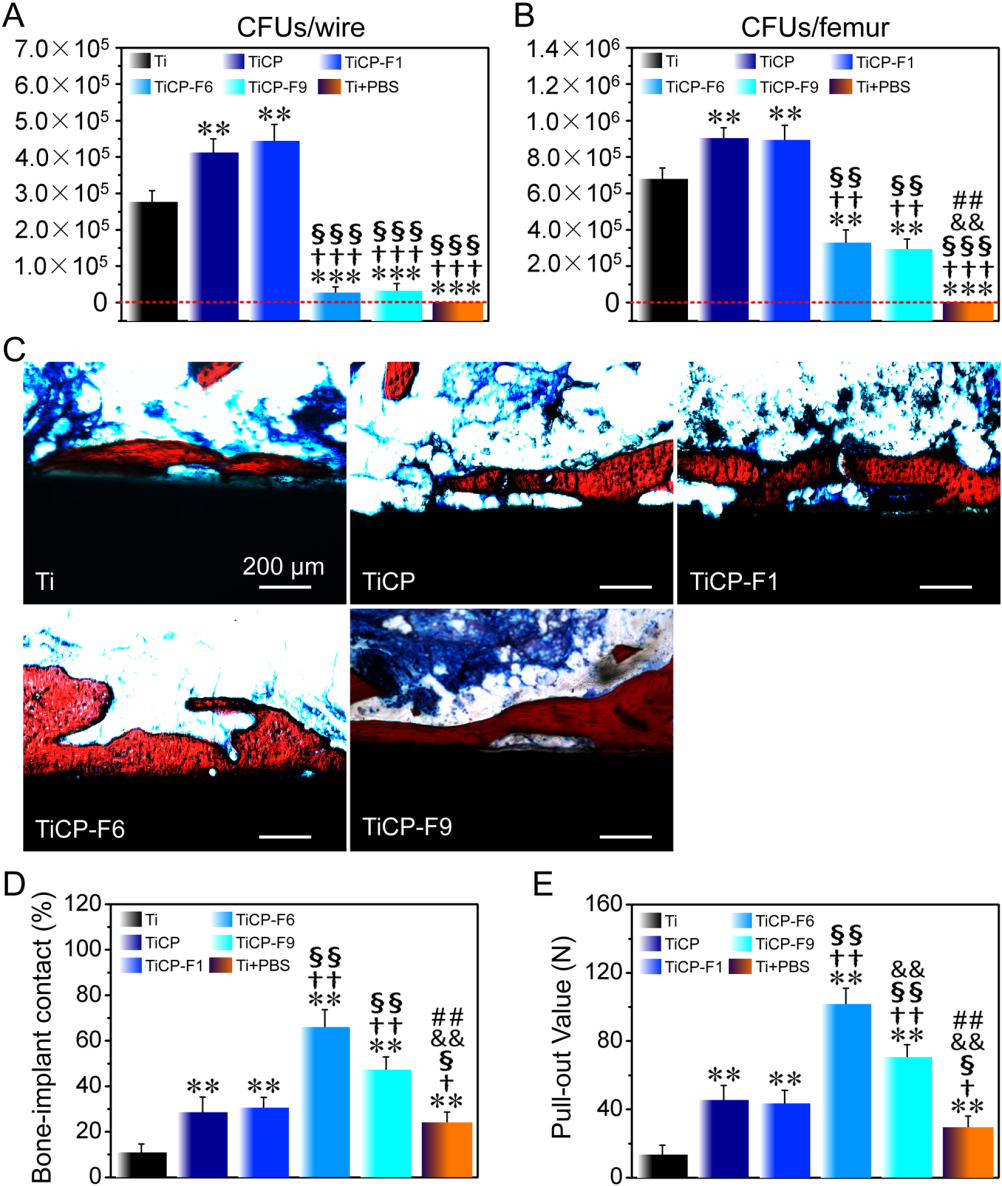
(**A**) Counting of the dislodged adhered bacteria after Ti and the coated wires rolling over SBA. (**B**) Amount of CFU in the wires surrounding femurs, quantified in pulverized bone from operated femur. (**C**) Histological observations of the implant/bone interface after 8 weeks of implantation in the infected rabbit model, where the tissues stained in red are the newly formed bone. (**D**) Percentage of bone-to-implant contact (BIC) and (**E**) pull-out force of the coated wires as well as Ti after 8 weeks of implantation. Data are presented as the means ± SD, n = 4. *p < 0.05 and **p < 0.01 compared to Ti; ^†^*p* < 0.05, ^††^*p* < 0.01 and ^†††^*p* < 0.001 compared to TiCP; ^§^*p* < 0.05, ^§§^*p* < 0.01 and ^§§§^*p* < 0.001 compared to TiCP-F1; ^&^*p* < 0.05 and ^&&^*p* < 0.01 compared to TiCP-F6; ^*##*^*p* < 0.01 compared to TiCP-F9.

Figure 7A,B show the amount of bacteria adhered on Ti, the coated Kirschner wires and in the surrounding femurs after 8 weeks of implantation in an infected rabbit model. The result indicted that the average colony forming units (CFUs) on the Kirschner wires followed the rank of TiCP≈TiCP-F1>Ti>TiCP-F6≈TiCP-F9, which were almost 0 on TiCP-F6 and TiCP-F9. Moreover, the average CFUs in femurs around the Kirschner wires followed the same trend. Especially, femurs around TiCP-F6 and TiCP-F9 coated Kirschner wires presented a small number of bacteria, although the wires themselves presented more bacteria. Totally, the *in vivo* antibacterial ability of the coatings was in good agreement with their *in vitro* antibacterial efficiency against *S. aureus*.

The *in vivo* osteogenesis of the coatings as well as Ti was also studied after 8 weeks of implantation in the same model. As histological stained images showing in Fig. 7C, all the coatings as well as Ti induced new bone formation on their surfaces but with different amount, presenting the rank of TiCP-F6>TiCP-F9>TiCP-F1≈TiCP>Ti. It’s worth noting that the new bone was separated from the surfaces of TiCP and TiCP-F1 as well as Ti by a thin layer of fibrous tissue, however, directly bond to the surfaces of TiCP-F6 and TiCP-F9 without discernible fibrous interval. Consequently, the osseointegration of the coatings as well as Ti evaluated by the bone-to-implant contact rate (Fig. 7D) and pull-out force (Fig. 7E) show the general trend of TiCP-F6>TiCP-F9>TiCP-F1≈TiCP>Ti. Moreover, new bone formation shows similar trend. All the results indicated that TiCP-F6 and TiCP-F9 induced better osseointegration than TiCP, TiCP-F1 and Ti in the infected rabbit model, and TiCP-F6 showed optimum osseointegration due to the effects of proper F addition on osteogenic and antibacterial activities.

## Discussion

It is widely accepted that the surface properties of a biomaterial including chemistry, energy/wettability, roughness and topography can influence its biological performance^23^. The different coatings fabricated on Ti in this study have the similar microporous feature, phase compositions of TiO_2_/calcium-phosphate, surface roughness and wettability. Therefore, one can assess the effects of different F amounts doped in the coatings without other influence factors.

In our previous study, the results showed that TiO_2_/calcium-phosphate coating incorporated with Sr, Co and F exhibits more excellent antibacterial and osteogenic activities compared to that incorporated with Sr and Co, indicating F incorporation could assign the coating with antibacterial and osteogenic abilities^10^. However, it has reported that overdose F ions inhibited the proliferation and osteogenic differentiation of osteogenesis-related cells^11,12^. Hence optimizing F incorporation dose in the TiO_2_/calcium-phosphate coating is essential. In the present study, TiCP and TiCP-F1 did not possess antibacterial activity against the colonization of *S. aureus* and *E. coli*, while TiCP-F6 and TiCP-F9 showed significant antibacterial activity. The results indicate that the incorporation of F into TiCP could enhance its antibacterial ability, which was positively related to the amount of incorporated F with an effective threshold of about 6 wt%. F can effective interference bacterial metabolism via a direct effect of an enzyme inhibitor and inhibit proton-translocating F-ATPases via forming metal-F complexes^25,26^. Figure 3 indicates that F ion can be released from doped TiCP. Furthermore, the XPS spectra in Fig. 2 display that Ti-F complex was formed in F-doped TiCP. Hence, the antibacterial activity of F-doped TiCP shall be ascribed to the F ion release and the formation of Ti-F complex.

Considering the similar surface topography, wettability, microstructure and phase compositions of TiCP, TiCP-F1, TiCP-F6 and TiCP-F9, the amount of F incorporation may be the reason for improving MSCs proliferation and osteogenic differentiation by TiCP-F6 and TiCP-F9. Actually, adding F into biomaterials was beneficial for cell proliferation and osteogenic differentiation^8,10,27^ with a dose-dependent manner^11,12,27^. In the present study, proliferation and osteogenic differentiation of MSCs were significantly enhanced with the increased F-doped amounts to 6 wt.%, and then reduced with continuous increase, due to side effects caused by overdose of F. Previous studies have similar results about the effects of doped-F amounts on osteoblasts, for instances, low amount of F ions was proved to stimulate proliferation and increase osteoblast differentiation, whereas high amount of F ions inhibited cell functions^28–30^. Thus, F ions could be released from the F-doped biomaterials and thereafter affect osteoblast behavior. In addition, Ca and P were found to improve the proliferation and differentiation of osteoblast, independently^31^, and this study shows advantages of Ca, P, and F ions on osteogenesis of MSCs other than osteoblasts.

Finally, *in vivo* antibacterial and osteogenic abilities of the coatings were also evaluated in the bacterium-infected rabbit model. The amounts of bacteria adhered on the coatings, Ti and in the surrounding femurs exhibited the same trend of TiCP≈TiCP-F1>Ti>TiCP-F6≈TiCP-F9. Furthermore, the new bone formation, ratio of BIC, and biomechanical strength of bone-implant integration all followed the rank of TiCP-F6>TiCP-F9>TiCP-F1≈TiCP>Ti (Fig. 7). The results showed that the TiCP-F6 and TiCP-F9 induced better antibacterial and osseointegration abilities than Ti, TiCP, and TiCP-F1, and TiCP-F6 showed optimum osseointegration due to the effects of proper F addition on osteogenic and antibacterial activities, keeping with the *in vitro* results of MSCs.

In conclusion, the microporous TiO_2_/calcium-phosphate coatings doped with F of tunable amount has been successfully developed on Ti by a simple MAO procedure. The microstructure, TiO_2_ phase composition, surface roughness, and wettability of the TiO_2_/calcium-phosphate coating were not obviously affected by the incorporation of F. All the coatings bond firmly to the Ti substrates and show enduring high adhesion strength with the Ca, P and F release in the biological environment. Both the *in vitro* and *in vivo* results demonstrated that the higher amount of F incorporation apparently improved antibacterial and osteogenic abilities of the coating, and the effects depended on the incorporated F amount. In the present study, TiCP-F6 gives rise to the best performance, inducing obviously enhanced dual osteogenic and antibacterial abilities with no significant cytotoxicity and hopefully contributes to an advanced implant of improved clinical performance.

## Methods

### MAO treatment of pure Ti

A bipolar pulse power supply was employed for the MAO treatment of Ti. The pure Ti disks (ϕ15 × 2 mm) and Ti Kirschner wires (ϕ2 × 10 mm) for *in vitro* and *in vivo* tests, respectively, were micro-arc oxidized in aqueous electrolytes of different chemical compositions (Table S1) by applying a positive/negative pulse voltage of 400 V with a pulse frequency of 100 Hz, and a duty ratio of 26% for 5 min. Ti samples treated by MAO were ultrasonically washed with alcohol and distilled water, and then dried at room temperature.

### Surface characterization

An X-ray diffraction (X’Pert PRO, The Netherlands) was used to identify the phase components of the coatings and an X-ray photoelectron spectroscopy (XPS; Axis Ultra, UK) was used to examine the elements and chemical species of the coatings. A field-emission scanning electron microscopy (FE-SEM; JEOL JSM-6700F, Japan), a surface contact-angle measurement machine (DSA30; KRUSS, Germany) and an atomic force microscopy (AFM; SPM-9500J3, Japan) were used to characterize the morphology of the coatings.

### Ion release and adhesion strength measurements of the coatings to the substrates

The coated samples were put into 10 ml PS solutions at 37 °C for 10, 20, 30, 40, 50 and 60 days successively. The concentrations of Ca, P and F ions released in the leaching liquid at pre-determined time points were measured by a inductively coupled plasma-mass spectrometry (ICP-MS; Nu Instruments, Wrexham, UK) and a F ion electrode (9409SC, Orion Research, UK) connected to an Ion Analyzer (901, Orion Research, UK), respectively. For the measurement of F ions, an ionic strength adjustment buffer (TISAB with CDTA) was applied, and a normalized curve with a standard F solution (F standard, Orion Research, UK) within a range of 0.1–20 ppm was obtained. Five samples (n = 5) were tested for each of materials.

Scratch tests of the coatings after immersion in PS solutions for different durations were conducted by applying an auto scratch coating tester and their adhesion strengths to the Ti substrates were evaluated. The critical load (Lc) defined as failure initiate was determined through the load versus acoustic output characteristics. The average value of the Lc was calculated by five replicate samples (n = 5).

### Protein adsorption assay

The coatings as well as pure Ti disks (as control) were employed as the samples for evaluation of protein adsorption. After incubation in α-MEM containing 10% FBS; for 24 h at 37 °C, the proteins adhered to the samples were detached by 1% sodium dodecyl sulfate (Solarbio). Then a NanoDrop 2000C device (Thermo Scientific, USA) was used to measure the protein adsorption at a wavelength of 280 nm. Five samples were tested for each of groups.

### Antibacterial activity evaluation

The antimicrobial effect of the coatings as well as Ti was evaluated by the bacterial counting method with *E. coli* (ATCC10536) and *S. aureus* (ATCC6538) as the gram-negative and the gram-positive representative, respectively. The method was described in detail in Supplementary Data.

### MSC harvest and culture

According to the ISO 10993-2:1992 animal welfare requirements and approved by the Institutional Animal Care and Use Committee (IACUC) of Xi’an Jiaotong University, MSCs were obtained from 1-week-old New Zealand rabbits^32^, as described in detail in Supplementary Data and 1 ml MSC suspension containing 2 × 10^4^ cells was seeded on the samples. All experiments were performed within passage 3.

### Cytotoxicity, cell adhesion, proliferation, and morphology

The activity of LDH released by the MSCs in the culture media was used as an index for the cytotoxicity of the coatings. After culturing of the MSCs on the coatings as well as Ti for 3 days, the culture medium was collected (among this period the medium was not changed) and centrifuged, and then the LDH activity in the supernatant was determined spectrophotometrically according to the manufacturer’s instruction (Sigma, USA).

The adhesion and proliferation of MSCs were evaluated by the CCK-8 assay after culturing on the coatings as well as Ti for 1 h, 5 h, 24 h, 3 days, 7 days, and 14 days.

After 3 days of incubation on the coatings as well as Ti, the MSCs were washed by PBS, fixed in 3% glutaraldehyde, and then dehydrated in series of ethanol followed by freeze-dried. Gold sputtering was performed on the samples prior to observation by the FESEM (JEOL JSM-6700F, Japan).

### Quantitative real-time PCR assay

The expressions of osteogenesis-related genes (Runx2, BSP, ALP, OPN, OCN, Col-I) of the MSCs, cultured on the coatings as well as Ti for 3, 7 and 14 days, were evaluated using real-time polymerase chain reaction. Isolated RNA (1 μg) via TRIzol reagent (Life Technologies, USA) from the MSCs was reversely transcribed into complementary DNA through a PrimeScrip RT reagent kit (TaKaRa, Japan). A quantitative real-time polymerase chain reaction detection system (Bio-Rad iQ5 Multicolor) with SYBRPremix ExTaqII (TaKaRa, Japan) was used to evaluate the expressions of the osteogenesis-related genes. An iQ5 Optical System (Bio-Rad, USA) with software version 2.0 was applied to analyze the data. Normalization for the expression levels of the target genes was carried out with the housekeeping gene glyceraldehyde-3-phosphate dehydrogenase (GAPDH). The primers for the target genes were listed in Table S3.

### Intracellular ALP activity and contents of specific proteins

By culturing on the coatings as well as Ti for 3, 7 and 14 days, washing with PBS for thrice followed by lysing with five standard freeze-thaw cycles in 0.1 vol% Triton X-100 (Life Technologies, USA) and shaken for 10 min was carried out for the cell-seeded samples. The intracellular ALP activity and intracellular contents of specific proteins (Col-I and OCN) in the cell lysates were evaluated with respective ELISA kits (Bluegene Ltd., China). The results were normalized to the total protein content of intracellular. Five samples were tested for each of groups (n = 5).

### Collagen secretion and ECM mineralization

Collagen secretion and ECM mineralization of the MSCs culturing on the coatings as well as Ti for 3, 7 and 14 days were assessed by the Sirius Red and Alizarin Red staining, respectively. The cell-seeded samples were stained by 0.1% Sirius Red (Sigma, USA) after washing with PBS and fixation to reveal the collagen, and mineralization was revealed by 40 mM Alizarin Red (pH 4.2, Sigma, USA). After washing with 0.1 M acetic acid or distilled water, the Sirius Red or Alizarin Red stain on the cell-seeded samples was dissolved in 0.2 M NaOH/methanol (1:1) or 10% cetylpyridinum chloride (Acros) to measure the optical density in quantity of 540 nm or 620 nm.

### *In vivo* osteogenic and antibacterial activities

According to the ISO 10993-2:1992 animal welfare requirements and approved by the Institutional Animal Care and Use Committee (IACUC) of Xi’an Jiaotong University, the animal experiments were carried out. Twenty-four adult (3 months in age) New Zealand male rabbits weighing 2–3 kg as objectives were used. The method was described in detail in Supplementary Data.

After 8 weeks of implantation, a measurement of bacterial adhesion on the coated Kirschner wires and Ti in the infected rabbit model was carried out, and they were immersed in PBS, sonicated, and vortexed to separate the bacteria, which were counted by the spread plate method mentioned above to draw CFUs. Measurement in the Kirschner wires surrounding femurs were also carried out. Femurs (n = 4) of each group were ground to powder under sterile conditions^33^ after snap freeze in liquid nitrogen. The powder of femurs for each of groups was vortexed in 2 ml PBS for 2 min. The supernatant was drawn for serial (10-fold) dilutions after centrifuging at 10,000 g for 15 s followed by analysis for CFUs in the wires surrounding femurs.

Then the bone/implant interface was histologically inspected by Van Gieson’s staining, the percentage of bone-to-implant contacts calculated based on the Van Gieson’s staining and the biomechanical strength of bone-implant integration measured by pull-out test^7^, as described in detail in Supplementary Materials.

### Statistical analysis

Three independent experiments were carried out to obtain data and a mean ± standard deviation (SD) was calculated. A SPSS 14.0 software (SPSS, USA) was applied to analyze the data. The level of significance was evaluated by a one-way ANOVA followed by a Student-Newman-Keuls *post hoc* test, and *p* < 0.05 and 0.01 were considered as significant and highly significant, respectively.

## Electronic supplementary material

Dataset 1

## References

[1] Horikawa T (2017). Retrospective cohort study of rough-surface titanium implants with at least 25 years’ function. Int J Implant Dent..

[2] Williams, D. F. Titanium and titanium implants, in: D. F. Williams (Ed.), Biocompatibility of Clinical Implant Materials, vol. 1, 9–44 (CRC Press, 1981).

[3] Qin H (2014). *In vitro* and *in vivo* anti-biofilm effects of silver nanoparticles immobilized on titanium. Biomaterials.

[4] Darouiche RO (2004). Treatment of infections associated with surgical implants. New. Engl. J. Med..

[5] Duffy GP, Berry DJ, Rowland C, Cabanela ME (2001). Primary uncemented total hip arthroplasty in patients b40 years old: 10- to 14-year results using first-generation proximally porous-coated implants. J Arthroplasty.

[6] Zhao L (2011). Antibacterial nano-structured titania coating incorporated with silver nanoparticles. Biomaterials.

[7] Yoshinari M, Oda Y, Kato T, Okuda K (2001). Influence of surface modifications to titanium on antibacterial activity *in vitro*. Biomaterials.

[8] Wang Y (2007). Osteoblastic cell response on fluoridated hydroxyapatite coatings. Acta Biomater..

[9] Birgani ZT, Gharraee N, Malhotra A, van Blitterswijk CA, Habibovic P (2016). Combinatorial incorporation of fluoride and cobalt ions into calcium phosphates to stimulate osteogenesis and angiogenesis. Biomed. Mater..

[10] Zhou JH, Zhao LZ (2016). Multifunction Sr, Co and F co-doped microporous coating on titanium of antibacterial, angiogenic and osteogenic activities. Sci. Rep..

[11] Shah FA (2016). Fluoride-containing bioactive glasses: Glass design, structure, bioactivity, cellular interactions, and recent developments. Materials Science and Engineering C.

[12] Gentleman E, Stevens MM, Hill RG, Brauer DS (2013). Surface properties and ion release from fluoride-containing bioactive glasses promote osteoblast differentiation and mineralization *in vitro*. Acta Biomate..

[13] Hu H (2012). Antibacterial activity and increased bone marrow stem cell functions of Zn-incorporated TiO_2_ coatings on titanium. Acta Biomater..

[14] Wei D, Zhou Y, Jia D, Wang Y (2007). Characteristic and *in vitro* bioactivity of a microarc-oxidized TiO_2_-based coating after chemical treatment. Acta Biomater..

[15] Liu W (2015). The *in vitro* and *in vivo* performance of a strontium-containing coating on the low-modulus Ti35Nb2Ta3Zr alloy formed by micro-arc oxidation. J. Mater. Sci. Mater. Med..

[16] Viornery C (2002). Surface modification of titanium with phosphonic acid to improve bone bonding: characterization by XPS and ToF-SIMS. Langmuir.

[17] Han Y, Chen D, Sun J, Zhang Y (2008). UV-enhanced bioactivity and cell response of micro-arc oxidized titania coatings. Acta Biomater..

[18] Yao ZQ (2010). Synthesis and properties of hydroxyapatite-containing porous titania coating on ultrafine-grained titanium by micro-arc oxidation. Acta Biomater..

[19] Chusuei CC, Goodman DW, Van Stipdonk MJ, Justes DR, Schweikert EA (1999). Calcium phosphate phase identification using XPS and time-of-flight cluster SIMS. Anal. Chem..

[20] Yu JC, Yu J, Ho W, Jiang Z, Zhang L (2002). Effects of F^−^ doping on the photocatalytic activity and microstructures of nanocrystalline TiO_2_ powders. Chem. Mater..

[21] Yu J, Xiang Q, Ran J, Mann S (2010). One-step hydrothermal fabrication and photocatalytic activity of surface-fluorinated TiO_2_ hollow microspheres and tabular anatase single micro-crystals with high-energy facets. CrystEngComm.

[22] Lord MS, Foss M, Besenbacher F (2010). Influence of nanoscale surface topography on protein adsorption and cellular response. Nano Today.

[23] Anselme K (2000). Osteoblast adhesion on biomaterials. Biomaterials.

[24] McBeath R, Pirone DM, Nelson CM, Bhadriraju K, Chen CS (2004). Cell shape, cytoskeletal tension, and RhoA regulate stem cell lineage commitment. Dev. Cell.

[25] Guha-Chowdhury N, Clark AG, Sissons CH (1997). Inhibition of purified enolases from oral bacteria by fluoride. Oral Microbiol. Immunol..

[26] Venkatesan K (2015). Structural and magnetic properties of cobalt doped iron oxide nanoparticles prepared by solution combustion method for biomedical applications. Int. J. Nanomedicine.

[27] Zhou JH, Li B, Zhao LZ, Zhang L, Han Y (2017). F-doped micropores/nanorods hierarchically patterned coatings for improving antibacterial and osteogenic activities of bone implants in bacteria-infected case. ACS Biomater. Sci. Eng..

[28] Rodriguez JP, Rosselot G (2001). Sodium fluoride induces changes on proteoglycans synthesis by avian osteoblasts in culture. J Cell Biochem.

[29] Farley JR, Wergedal JE, Baylink DJ (1983). Fluoride directly stimulates proliferation and alkaline phosphatase activity of bone-forming cells. Science.

[30] Song XD, Zhang WZ, Li LY, Pang ZL, Tan YB (1988). The effect of sodium fluoride on the growth and differentiation of human fetal osteoblasts-an *in vitro* study. Fluoride.

[31] Ma S (2005). Effects of dissolved calcium and phosphorous on osteoblast responses. J Oral Implantol.

[32] Sakai D (2003). Transplantation of mesenchymal stem cells embedded in Atelocollagens gel to the intervertebral disc: a potential therapeutic model for disc degeneration. Biomaterials.

[33] Lucke M (2003). Gentamicin coating of metallic implants reduces implant-related osteomyelitis in rats. Bone.

