# Supplementary material for: F-doped TiO2 microporous coating on titanium with enhanced antibacterial and osteogenic activities
Source: Sci Rep. 2018 Dec 14;8:17858. doi: 10.1038/s41598-018-35875-6 (PMC6294799; doi:10.1038/s41598-018-35875-6)
Supplement: Supplementary file 1 — Dataset 1 [file 41598_2018_35875_MOESM1_ESM.pdf]

F-doped TiO_2 microporous coating on titanium with enhanced antibacterial and osteogenic activities

Jianhong Zhou [1,2], Bo Li [2] and Yong Han [2,*]

[1] *Institute of Physics & Optoelectronics Technology, Baoji University of Arts and Sciences, Baoji, 721016, China*

[2] *State Key Laboratory for Mechanical Behavior of Materials, Xi'an Jiaotong University, Xi'an, 710049, China*

**Corresponding author at: State Key Laboratory for Mechanical Behavior of Materials, Xi'an Jiaotong University, Xi'an, 710049, China*

E-mail addresses: yonghan@mail.xjtu.edu.cn (Yong Han)

Methods

Antibacterial activity evaluation

The bacteria were grown overnight in Luria–Bertani (LB) medium containing 10 g L^{-1} peptone, 5 g L^{-1} NaCl, and 5 g L^{-1} beef extract. The pH was adjusted to be between 7.0 and 7.2 using 1 M NaOH solution. *S. aureus* and *E. coli* were then re-suspended in phosphate buffered saline (PBS) at a concentration of 10^7 and 10^6 cells/ml (calibrated in terms of colony forming units (CFU) using the spread plate method), respectively. One ml of the bacterial suspension discussed above was added to each sample and incubated for 24 h at 37°C. Then the bacteria on the samples were dissociated, collected and inoculated into a standard agar culture medium. After incubation at 37°C for another 24 h, the live bacteria were counted in accordance with the National Standard of China GB/T 4789.2 protocol. The antibacterial ratio was calculated using the formula, A.R.= (A-B)/A×100%, where A.R. means the antibacterial ratio, A is the average number of bacteria on the control specimen, the flat Ti (CFU per specimen), and B is the average number of bacteria on the testing specimen (CFU per specimen).

In the FESEM examination, 1 ml of the bacterial suspension discussed above was inoculated to each Ti sample and incubated for 24 h at 37°C. The samples were fixed, dehydrated in a series of ethanol solutions for 30 min each followed by the final dehydration conducted in absolute ethanol twice, dried in the hexamethyldisilizane ethanol solution series, and finally observed with FESEM.

MSC harvest and culture

The animal experiments were conducted according to the ISO 10993-2:1992 animal welfare requirements and approved by the Institutional Animal Care and Use Committee (IACUC) of Xi'an Jiaotong University. Briefly, bone marrow was aspirated from the femora and tibias, from which the mononucleated cells were isolated via density gradient centrifugation. The cells obtained were plated in cell culture flasks containing 20 ml of a-MEM containing 10% FBS and 1% antibiotics, and cultured at 37 °C in a humidified atmosphere of 5% CO_2 and 95% air. Non-adherent cells were removed and the adherent cells were collected for further expanding.

***In vivo* osteogenic and antibacterial activities**

The animal experiments were conducted according to the ISO 10993-2:1992 animal welfare requirements and approved by the Institutional Animal Care and Use Committee (IACUC) of Xi'an Jiaotong University. Twenty-four adult New Zealand male rabbits 3 months in age weighing 2-3 kg were used. *S. aureus* was chosen to create osteomyelitis. After intraperitoneal injection of 4% chloral hydrate (0.9 ml/100 g body weight) and sterilization with povidone iodine, 4 holes of 1 cm distance from each other were sequentially drilled using a disinfected hand-operated drill (2 mm in diameter) on the left femur of rabbit. Four Ti or the coated Kirschner wires were classificatorily implanted into the drilled holes of each femur, as shown in Schematic 1A. Subsequently, 20 µl of the PBS-diluted suspension of *S. aureus* with a density of 10^5 CFU/ml was injected into the medullary cavity with a microsyringe to create an

infected model. The Ti Kirschner wire together with 20 μl PBS injected into the medullary cavity (denoted as Ti+PBS) was set as a control. After bacterial inoculation, the fascia and skin were sutured. Following surgery, the rabbits were housed in the separated cages and allowed to eat and drink ad libitum up to weeks 8 when they were sacrificed by intraperitoneal injection of overdose pentobarbitone sodium.

Immediately thereafter killing the rabbits, the femurs containing implants were fixed in neutral buffered formalin, dehydrated by ascending concentrations of ethanol, and finally embedded in polymethylmethacrylate (PMMA). The embedded specimens were cut into 150 μm thick sections perpendicular to the bone long axis using a saw microtome (Leica SP1600, Hamburg, Germany), ground, and polished to a final thickness of about 40 μm. The sections were stained with Van Gieson's picrofuchsin and examined microscopically to visualize the mineralized bone tissue (red). The images were captured with a fluorescence microscope (Olympus IX 71, Olympus, Japan) and the panoramic images were acquired by Multiple Image Alignment (MIA), an advanced image capturing process enabled by cellSens Dimension software, and analyzed using Image-ProPlus software. Histometric analysis for evaluating the percentage of bone-to-implant contact (BIC) was performed on 4 sections on each embedded specimen.

The biomechanical pull-out test was used to assess the strength of bone-implant integration. The femurs containing implants (n=3 for each group) were harvested after 8 weeks of implantation in the infected rabbit model , which were partially embedded in PMMA with the implants' top being horizontal. The testing machine (Shimadzu, AGS-10kNG, Japan) was used to pull the implant vertically out at a cross-head speed of 1 mm/min. The load-displacement curve was recorded and the maximum pull-out

force was then calculated.

Table S1. The corresponding MAO electrolyte compositions, and elemental composition of the coatings detected by XPS.

Coatings	Aqueous electrolyte concentration (M)			Elemental composition (wt.%)				
	Calcium acetate	β-glycero phosphate disodium	Sodium fluoride	Ti	O	Ca	P	F
TiCP	0.05	0.02	-	45.7±0.5	44.3±0.6	5.7±0.3	4.3±0.5	-
TiCP-F1	0.05	0.02	0.05	44.9±0.8	43.5±0.7	6.1±0.4	4.4±0.3	1.1±0.2
TiCP-F6	0.05	0.02	0.15	43.6±0.6	40.2±0.9	5.9±0.5	4.6±0.4	5.7±0.5
TiCP-F9	0.05	0.02	0.30	42.3±0.8	38.2±0.7	6.2±0.6	4.1±0.4	9.2±0.3

Table S2. Roughness values, and contact angles of the coatings.

Coatings	Roughness (nm)			Contact angle (deg.)
	Ra	RMS	Rz	
TiCP	456.9±43.7	461.5±40.6	1192.3±141.5	47.6±3.5
TiCP-F1	465.6±40.8	468.6±39.2	1208.7±138.2	46.5±4.1
TiCP-F6	459.3±39.2	476.5±37.5	1213.5±136.7	45.8±3.9
TiCP-F9	468.5±41.5	473.4±36.9	1201.8±143.8	43.9±4.8

Table S3. Primers Used for qRT-PCR.

Gene	Forward primer sequence (5′-3′)	Reverse primer sequence (5′-3′)
Runx2	TGGTGTTGACGCTGATGGAA	ATACCGCTGGACCACTGTTG
BSP	GTCAGAACTGCTGGGACTCG	TGGCATTAGGTGTACTTGACAGT
ALP	CTGAGCGTCCTGTTCTGAGG	GTTCCTGGGTCCCCTTTCTG
OPN	GTGTACCCCACTGAGGATGC	CACGTGTGAGCTGAGGTCTT
OCN	CTTCGTGTCCAAGAGGGAGC	CAGGGGATCCGGGTAAGGA
Col-I	TGCAGGGCTCCAATGATGTT	TGCAGGGCTCCAATGATGTT
GAPDH	ATCAAGTGGGGTGATGCTGG	TACTTCTCGTGGTTCACGCC
